# A trimetallic organometallic precursor for efficient water oxidation

**DOI:** 10.1038/s41598-019-40236-y

**Published:** 2019-03-06

**Authors:** Sepideh Madadkhani, Reza Babadi Aghakhanpour, Jitendra Pal Singh, Robabeh Bagheri, Keun Hwa Chae, Zhenlun Song, Mohammad Mahdi Najafpour

**Affiliations:** 1 Department of Chemistry, Institute for Advanced Studies in Basic Sciences (IASBS), Zanjan, 45137-66731 Iran; 2 Advanced Analysis Center, Korea Institute of Science and Technology (KIST), Seoul, 02792 Republic of Korea; 3 Surface Protection Research Group, Surface Department, Ningbo Institute of Materials Technology and Engineering, Chinese Academy of Sciences, 519 Zhuangshi Road, Ningbo, 315201 China; 4 Center of Climate Change and Global Warming, Institute for Advanced Studies in Basic Sciences (IASBS), Zanjan, 45137-66731 Iran; 5 Research Center for Basic Sciences & Modern Technologies (RBST), Institute for Advanced Studies in Basic Sciences (IASBS), Zanjan, 45137-66731 Iran

## Abstract

Herein, we report an iron/nickel/zinc mixed oxide as a catalyst for the electrochemical water oxidation. This catalyst was synthesized by a straightforward method for the synthesis of an iron/nickel/zinc mixed oxide through the calcination of a Fe/Ni/Zn organometallic compound. The calcined product contains Fe and Ni as crucial ions for water oxidation, accompanied by the presence of Zn ions. The removal of Zn ions from the mixed oxide provides more active sites on the surface of the catalyst. The composition of the compound was characterized by some common methods and found to be an efficient water-oxidizing catalyst. The catalyst on FTO at pH = 13 yields a current density of 12 mA/cm^2^ at 1.2 V (vs. Ag│AgCl). After 5 hours at 1.1 V, the electrode not only shows no decrease in performance, but also shows an increase from 4 to 7 mA/cm^2^ in the water oxidation activity. Tafel plot, for the electrode at pH = 13 in KOH solution (0.1 M) showed linearity for the graph of lg j vs. V with both relatively low (220.4 mV per decade) and high overpotentials (903.7 mV per decade).

## Introduction

Switching from fossil fuels to renewable energies is an inevitable task that should be performed in the near future to provide clean and sustainable energies for human beings. However, many renewable kinds of energy are too intermittent to be used at a large scale; thus a large capacity for the energy storage is necessary^1^. Water splitting toward hydrogen production is a promising approach to store such energies^2^. In this regard, there are some bottlenecks for water splitting systems. Among different solutions, electrocatalytic water oxidation process is considered to be a breakthrough in water-splitting technology^3^.

It has been proved that first-row transition metal-based water-oxidizing catalysts can be appropriate catalysts in water-splitting systems. In particular, V, Cr, Mn, Fe, Co, Ni, and Cu elements were significantly employed in the presence of cerium(IV) ammonium nitrate or Ru(bpy)_3_^3+^ as chemical oxidants, under the electrochemical, photochemical or electro-photochemical conditions^4–8^. Under the electrochemical conditions, current densities (j) higher than 1 mA/cm^2^ at low overpotentials are necessary for water oxidation^9^. Among the metal oxides, Ni/Fe (oxy)hydroxide, which can be synthesized with different morphologies, stoichiometries and crystallinities, are able to catalyze the water-oxidation reaction in alkaline electrolyte solutions. Also, it is worthy to note that Ni/Fe-based oxides are efficient, stable, low-cost and environmentally friendly. Additionally, some research groups previously reported that Ni/Fe (oxy)hydroxides have the lowest overpotential toward water oxidation under the alkaline conditions^10–14^.

Ni/Fe (oxy)hydroxides are used as the precursors to synthesize the other phases of Ni/Fe oxides^15^, nitrides^16^ and sulfides^17^. These oxides also are applied in hydrogen evolution and photoelectrochemical water splitting^18,19^. In this context, Corrigan *et al*. firstly investigated the catalytic activity of Ni/Fe (oxy)hydroxides for water oxidation in the 1980s^20–22^. The synergic effect between Ni and Fe oxide for water oxidation was reported in 1987^22^. The role of Fe ions is still unknown, however, as reported earlier, the water oxidation overpotential decreases when the amount of Fe increases from 0 to 10% in order to reach a minimum at the range of 10% and 50%^23,24^. Ni/Fe (oxy)hydroxides have also been employed in the development of an electrolyzer having a voltage below 1.5 V with a single AAA battery^25^ and high-performance rechargeable Zn–air batteries^26^.

In the case of stability, the Pourbaix diagram of nickel shows that at pH ˃9, Ni(II) oxide is the dominant stable form of Ni^27^ and Ni(II) oxide at large positive bias is also stable^28^.

Organometallic compounds have widely been employed as a reagent, pre-catalyst or true catalyst for water oxidation. In the present investigation, we used a Fe/Ni/Zn organometallic compound (**1**) as a precursor to synthesize Fe/Ni/Zn oxide. As discussed earlier, Fe impurity in the nickel hydroxide is one of the most promising discoveries in water oxidation which significantly increases the amount of water oxidation^22^. In addition to Ni and Fe as important ions for water oxidation, the present organometallic compound includes Zn ion as an amphoteric ion which can be removed in the presence of KOH solution to obtain more active sites on the surface of the catalyst and thus a self-improvement is observed during reaction.

## Results

Ferrocene is a very famous organometallic compound with a highly reversible oxidation to form the corresponding cation in many organic solvents^29^. It was reported that diphenylphosphine moieties on cyclopentadienyl rings of dppf (1,1′-Bis(diphenylphosphino)ferrocene) make the oxidation of iron to be more difficult than ferrocene in 1,2-dichloroethane solvent^30–32^. In this way, **1** has indicated irreversible anodic peaks in cyclic voltammetry (CV) which are attributed to a decomposition reaction in acetonitrile (Fig. S2a) (ESI†). The peak at around 0.35 V is attributed to the Fe(II) to Fe(III) oxidation which is highlighted by gray in Fig. S2a (ESI†). This peak becomes clearer in the square wave voltammograms (SWV) which is highlighted by yellow Fig. S2b (ESI†). Also, the peaks observed at around 1 and 1.5 V are attributed to the decomposition of **1**. On the other hand, the electrochemical properties of **1** are completely different in the presence of small amount of water compared with pure acetonitrile (Fig. S2c (ESI†)). As shown in Fig. S2c (ESI†), in CVs of **1** with higher scan rates, the peak for the decomposition reaction at 1.4 V is followed by a sharp peak for water oxidation. SWV analysis vividly indicated the peak related to the decomposition reaction (Fig. S2d (ESI†)). In the following, the amperometry test of the complex **1** revealed that the decomposition reaction occurs at the first seconds of the reaction (Fig. S2e (ESI†)). Also, the continued cyclic voltammetry confirmed that at 1.0 V, a decomposition reaction happened Fig. S2f,g (ESI†). Using the spectroelectrochemistry technique, a broad peak was observed at around 380 nm, which is related to a new entity, generated from the decomposition process (Fig. S2h (ESI†). All the obtained data proved that the complex **1** is not stable under the water-oxidation conditions.

In the next step, the complex was calcined to obtain a mixed oxide. As shown in the thermal gravimetric analysis (TGA) diagram (Fig. S3, ESI†), at the temperatures higher than 500 °C, organic moieties of the complex are completely removed and a mixed oxide is formed. Finally, Fe/Ni/Zn oxide was treated by KOH (1.0 M) for 5 hours to obtain the final catalyst.

**Figure 1:**
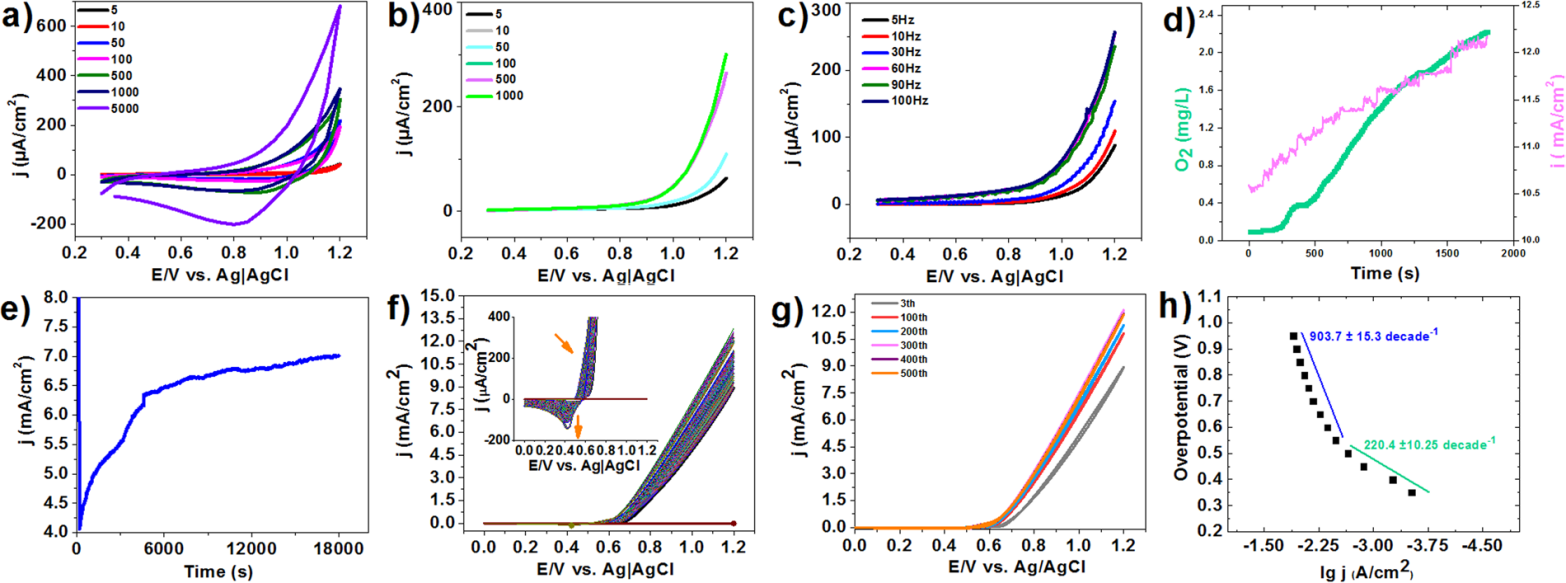
CV (**a**), LSV (**b**) and SWV (amplitude: 25 mV) (**c**) of Fe/Ni/Zn oxide at different scan rates in LiClO_4_ (0.25 M). Oxygen evolution/amperometry (1.2 V, KOH: 0.1 M) for Fe/Ni/Zn oxide (**d**). Amperometry for Fe/Ni/Zn oxide in KOH (0.1 M) at 1.1 V (**e**). Continuous CVs of Fe/Ni/Zn oxide at 100 mV/s in KOH (0.1 M) at (**f**,**g**). Tafel plot for Fe/Ni/Zn oxide in KOH (0.1 M) (**h**). 30 μL of oxide was dispersed in water (1.0 mg/mL) and dripped on the FTO electrode and dried at 60 °C. Then, 10 µL of 0.5 wt% Nafion solution was cast on the surface of the FTO electrode (1.0 cm^2^).

For the Fe/Ni/Zn mixed oxide, CVs with different scan rates are shown in Fig. 1a. No peak was observed at pH 7 in cyclic voltammetry (CV), linear sweep voltammetry (LSV), and Square wave voltammetry (SWV) (Fig. 1a–c). The experiments showed that the obtained oxide could be an efficient catalyst for water oxidation at high pH values (Fig. 1d–h).

20 mL of an argon saturated KOH (0.1 M) solution at room temperature was used as the electrolyte solution for water oxidation. The catalyst on FTO at pH = 13 yields a current density of 12 mA/cm^2^ at 1.2 V (all potential in the paper was reported vs. Ag│AgCl) (Fig. 1d). At the around overpotential of 850 mV, the catalyst showed no decrease in the amount of water oxidation. Conversely, an increase was observed for the electrode (7 mA/cm^2^) **(**Fig. 1e**)**, indicating a long-term stability and good performance. Long-time amperometry also indicated an increase in the current density (j). Continued CVs showed that the water-oxidizing activity of the compound increased during the cyclic voltammetry process **(**Fig. 1f,g**)**. For the catalyst, before the water oxidation potential, a peak was observed which may be attributed to “Ni(II) to Ni(III) oxidation”, according to the Pourbaix diagram^27^.

lg (j/Acm^−2^) vs. overpotential (Tafel plot) was plotted for the mixed oxide on FTO electrode in KOH solution (0.1 M) (Fig. 1h). Tafel plot of the electrode at pH = 13 in KOH solution (0.1 M) showed a linearity for the lg j vs. potential with two slopes corresponding to both relatively low (220.4 mV per decade) and high overpotentials (903.7 mV per decade) (Fig. 1h). The smaller Tafel slope at low overpotentials for the catalyst indicates its more favorable kinetics in comparison to many catalysts^33,34^. Low Tafel slope is often influenced by electron and mass transport. It can be concluded that electron and mass transport are easily performed for the catalyst at least at lower overpotentials. Moreover, the structure of Ni/Fe at nanoscales may be important for increasing the efficiency of the catalyst, particularly at lower overpotentials. Table S1 shows the catalytic performance of of some oxido-based material^35^.

**Figure 2:**
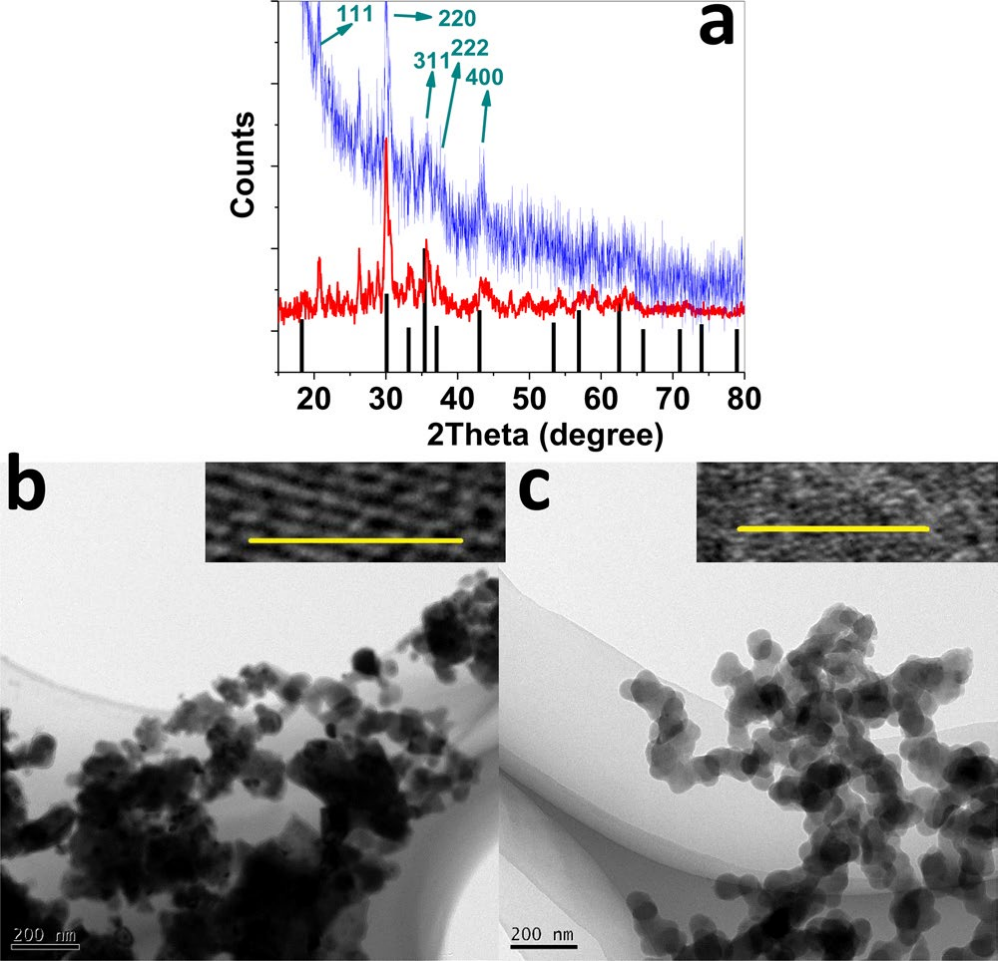
XRD patterns before (red) and after (blue) KOH treatment (**a**). (HR)TEM before (**b**) and after (**c**) KOH treatment. Scale bar for b and c is 5.0 nm.

Before and after KOH treatment, X-ray powder diffraction (XRD) characteristic weak peaks for (Ni, Zn)Fe_2_O_4_ (JCPDS CARD: 00-008-0234) were detected (Fig. 2a), but amorphization occurred after the water oxidation. A very broad peak at 18.7° was attributed to the (111) plane.

HRTEM images showed irregular layers with d-spacing of 0.45–0.5 nm, which are attributed to the (111) plane (Fig. 2b,c; Figs S4 and S5, ESI†). In a few areas, the d-spacing of 0.3 nm for the (220) plane was also observed. The (220) planes are completely regular compared with the (111) planes. Additionally, some defects and also amorphization were observed for (111) planes. Although the amorphization occurred after KOH treatment, some crystalline structures were observed in a few areas.

**Figure 3:**
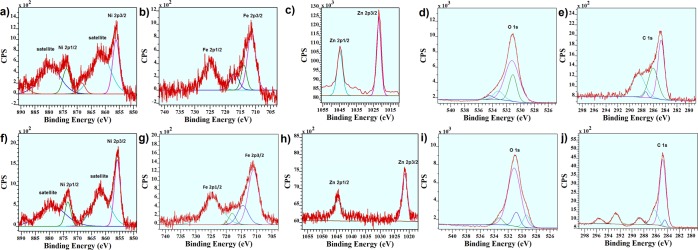
XPS spectra for Fe/Ni/Zn oxide before (**a**,**b**) and after (**c**,**d**) KOH treatment.

The XPS results before and after the KOH treatment revealed the presence of carbon, oxygen, iron, zinc, and nickel on the surface of the oxide (Fig. S6, ESI†). Ni2p_3/2_ for the oxide before and after the KOH treatment showed a peak at 856 eV (Fig. 3a,f), which is attributed to the Ni(II) entity^36^. The peaks of iron for the oxide before and after KOH treatment exhibited peaks at ~711 and 725 eV, attributed to the Fe(III) ion (Fig. 3b,g)^37^. Zn2p_3/2_ for the oxide before and after the KOH treatment showed a peak at 1021.7 eV (Fig. 3c,h), which is attributed to the Zn(II). Following the XPS analysis, the Ni/Fe oxide on the surface of the FTO electrode is suggested to be a true catalyst under these conditions. However, the ratio of Ni/Zn increased from 0.8 to 1.4, which similar to EDX-SEM indicates the removing Zn under the alkaline condition. Since Zn ions are amphoteric, a suggestion is that the removal of these ions in KOH solution. O1s for the catalyst before and after the KOH treatment showed no significant changes (Fig. 3d,i), but C 1s area showed that after treatment the oxidized carbon from the ligand decomposition was removed from the catalyst after KOH treatment (Fig. 3e,j).

**Figure 4:**
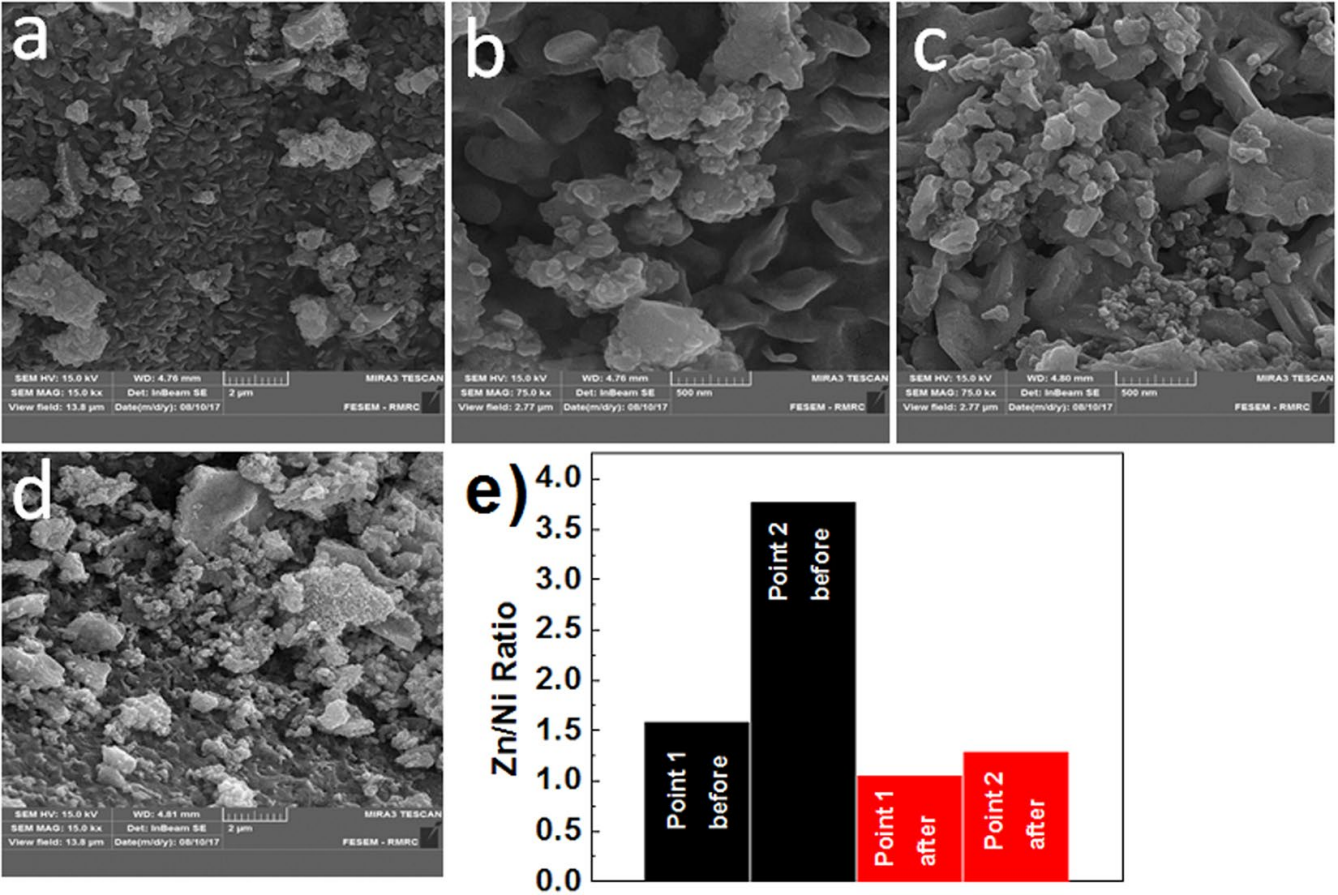
SEM images from for Fe/Ni/Zn oxide before (**a**,**b**) and after (**c**,**d**) water oxidation. The ratio of Zn:Ni for Fe/Ni/Zn oxide before (black) and after (red) water oxidation for both points (**e**).

SEM images before and after water oxidation displayed nanosized particles with no special morphologies (Fig. 4; Figs S7–S10, ESI†). EDX-SEM after water oxidation showed a lower ratio of Zn:Ni in the structure of the electrode (Fig. 4; Figs S7–S10, ESI^†^). Thus, a hypothesis is that the removing Zn ions provides more active sites on the surface of the catalyst and results in a self-improvement for the catalyst. Therefore, KOH not only acts as an electrolyte and substrate but also removes the amphoteric Zn ions. On the other hand, the amorphization was observed for the catalyst after the reaction.

## Discussion

**Figure 5:**
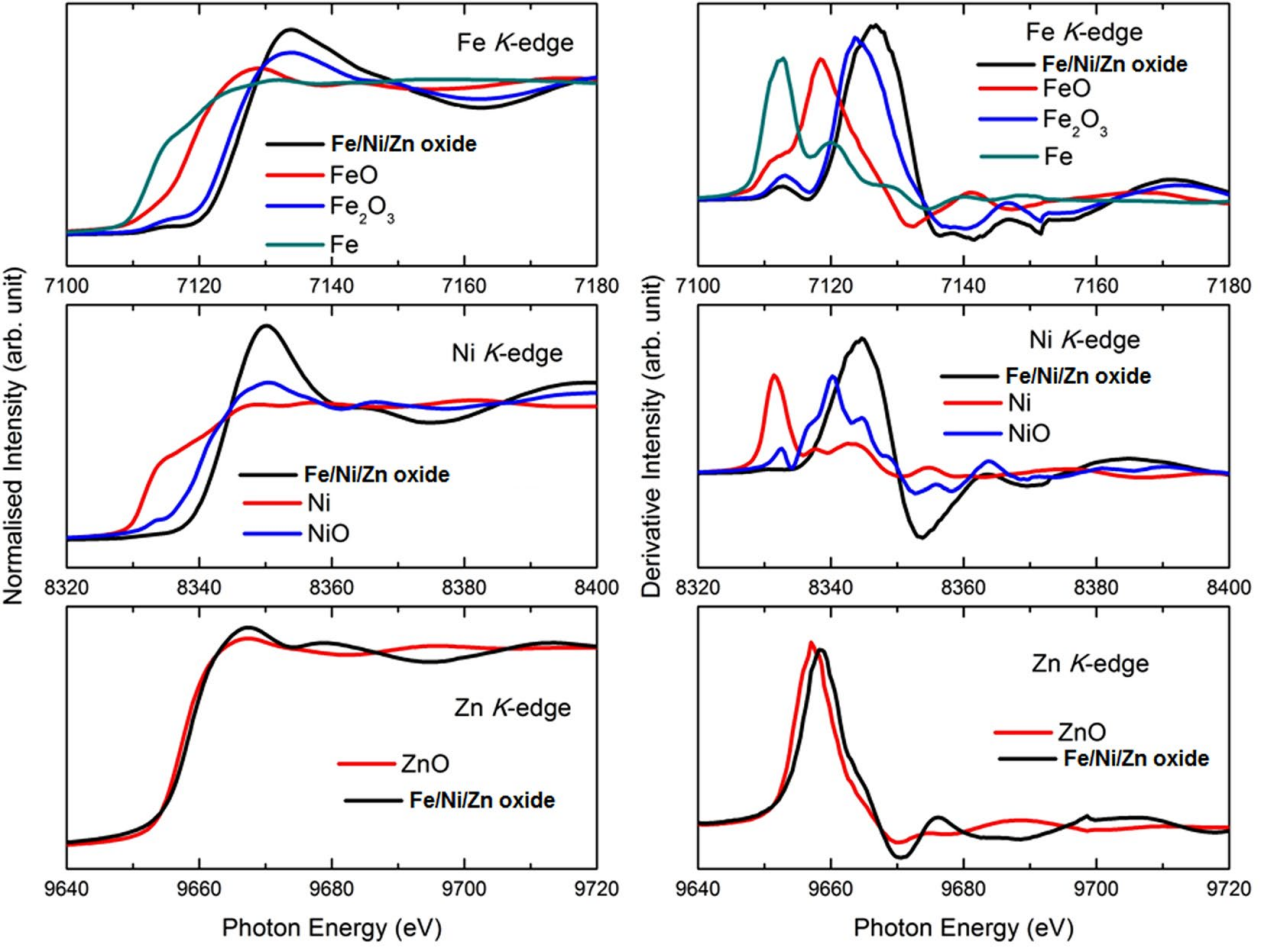
XANES and derivative spectra of Fe/Ni/Zn oxide at Fe, Ni and Zn K-edges.

The XANES measurements shown in Fig. 5 speculate the valence state of different ions and the local environment information. These spectra showed that the main edge of Fe/Ni/Zn oxide after the KOH treatment lies above the metal edges indicating the oxidation of various elements in the samples. X-ray absorption near edge structure (XANES) spectra indicated that the Fe present in Fe/Ni/Zn oxide has a mixed (II and III) valence state. Seemingly, Ni has an oxidation state higher than II while the valence state of Zn is equal to II.

**Figure 6:**
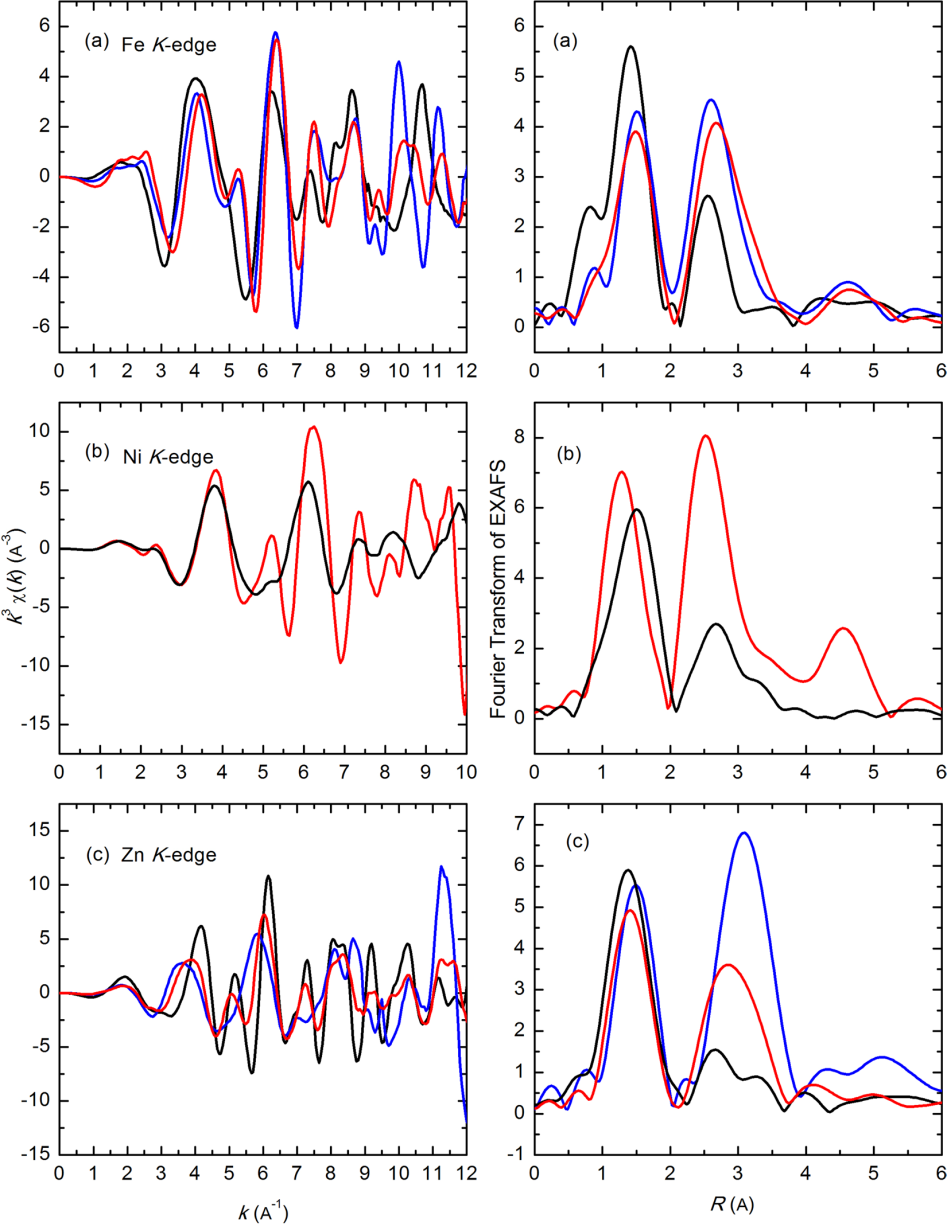
k^3^-EXAFS spectra at Fe, Ni and Zn K-edges of Fe/Ni/Zn oxide (black), (Ni,Zn)Fe_2_O_4_ (red) and ZnFe_2_O_4_ (blue) (left). Fourier Transform of EXAFS spectra at Fe, Ni and Zn K-edges of Fe/Ni/Zn oxide (black) and (Ni,Zn)Fe_2_O_4_ (red) and ZnFe_2_O_4_ (blue) (right).

The local environment of these ions are not similar to corresponding simple oxides such as NiO, Fe_2_O_3_, Fe_3_O_4_, FeO and ZnO as the *K*-oscillations are quite different (Fig. S12, ESI†). As XRD reveals that structure of Ni/Zn/Fe oxide is analogues to that of (Ni,Zn)Fe_2_O_4_, hence, the EXAFS spectra were compared with (Ni,Zn)Fe_2_O_4_ and ZnFe_2_O_4_ to establish atomic structure (Fig. S12, ESI†). *K*-weight EXAFS spectra and its Fourier Transform (FT) of the materials are shown in Fig. 6. *K*-weight EXAFS spectra revealed the different nature of oscillations than the reference compounds, which envisages the change of local atomic structure because of the amorphization. FT of EXAFS spectra for Fe/Ni/Zn oxide exhibit the presence of metal-oxygen interaction; however, the weak metal-metal interaction compared to the reference compound is also observed.

**Figure 7:**
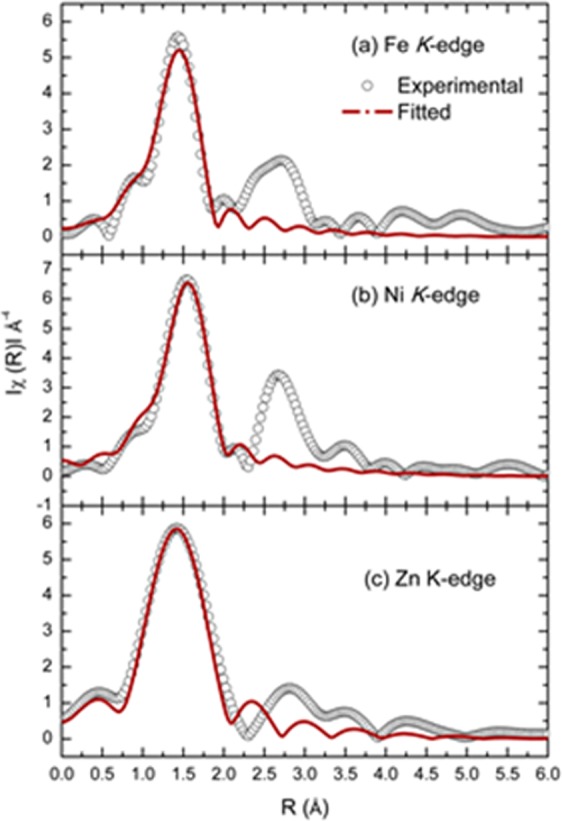
Fourier Transform of simulated and experimental EXAFS spectra at Fe, Ni and Zn K-edges of Fe/Ni/Zn oxide using quick first shell fit.

To get more details on the quantitative description of metal-oxygen bond lengths and the local coordination of metal ions, EXAFS spectra of Fe/Ni/Zn oxide were simulated using quick first shell fit. The simulated spectra are shown in Fig. 7. In these spectra, it is clear that the first oscillation is well fitted by the considering metal-oxygen bonds in the Fe/Ni/Zn oxide. The coordination numbers and metal-oxygen bond lengths are tabulated in Table 1.

**Table 1:** Co-ordination (N), radial distance (R), Debye-Waller factor (σ^2^) and correction to the main edge (ɛ) and R-factor obtained from the simulation at Fe K, Ni K and Zn K-edges.

Metal edge	N	R (Å)	σ^2^ (Å^2^)	ɛ (eV)	R-factor
Fe	3.9 ± 0.5 (6)	1.936 ± 0.005 (2.05)	0.009 ± 0.002	−7.62	0.01
Ni	5.7 ± 0.8 (6)	2.03 ± 0.01 (2.09)	0.010 ± 0.002	−3.2	0.01
Zn	3.9 ± 1.6 (4)	1.956 ± 0.006 (1.965)	0.004	−5.3	0.04

Values in parenthesis are given for Fe-O in Fe_2_O_3_, Ni-O in NiO and Zn-O in ZnO.

It is worthy to note that, both **1** and Fe/Ni/Zn oxide were not active the water oxidation in the presence of cerium(IV) ammonium nitrate as a chemical oxidant (Fig. S13, ESI†).

## Conclusions

In conclusion, we reported a new procedure to synthesize an efficient Fe/Ni/Zn-based water-oxidizing catalyst by the thermal decomposition of a trimetallic organometallic compound. On the basis of the obtained results, a structure similar to (Ni, Zn)Fe_2_O_4_ is proposed. The obtained nanosized oxide on FTO at pH = 13 showed a current density of 12 mA/cm^2^ at 1.2 V. The catalyst also showed a good long-term stability and performance. Since Zn ions are amphoteric, they can be soluble in KOH solution and results in a self-improvement for the catalyst. Removing the Zn ions from the NiFeZnO_x_ structure may be a promising method to increase the catalytic activity of the present oxide. Tafel plot, for the electrode at pH = 13 in KOH solution (0.1 M) showed linearity for the graph of lg j vs. V with both a relatively low (220.4 mV per decade) and a high overpotentials (903.7 mV per decade).

## Methods

All reagents and solvents were purchased from the commercial sources (Merck and Sigma-Aldrich Companies) and were used without any further purification. **1** was purchased from Sigma-Aldrich Company (NMR data in CDCl_3_: δ(^1^H) = 2.06 [d, 2 H, ^2^*J*_HH_ = 6.02 Hz, H_2_C-Ni]; 4.25, 435, 4.83. 530 [m, 8 H, overlapping protons of Cp rings]; 4.42 [d, 1 H, ^4^*J*_HH_ = 6.55 Hz, = CH adjacent to phenyl ring]; 5.90 [dt, 1 H, ^3^*J*_HH_ = 6.55 Hz, ^3^*J*_HH_ = 6.02 Hz, = CH adjacent to Ni]; 7.00–7.96 [overlapping protons of phenyl moiety and phenyl rings of PPh_2_]). The electrochemical experiments were performed using an EmStat^3+^ from PalmSens (Netherland). A three-electrode system includes an FTO slide, Pt foil as counter and Ag|AgCl|KCl_sat_ as reference electrodes were applied for the investigation.

LiClO_4_ (0.25 M) or KOH (0.1 M) was used as electrolyte. X-ray photoelectron spectroscopy (XPS) measurements were done on an X-ray BesTec XPS system (Germany) with an AlK_α_ X-ray source (hυ = 1486.6 eV). Scanning electron microscope (SEM) was carried out with a Philips CM120. For high-resolution transmission electron microscopy (HRTEM and TEM), the samples were studied using an LEO 1430VP. X-ray powder diffraction patterns were recorded by a Bruker D8 ADVANCE (Germany) diffractometer (CuK_α_ radiation). Experimental details of extended X-ray absorption fine structure (EXAFS) measurements for Fe/Ni/Zn oxide are measured at 1D KIST beam-line at Pohang Accelerator Laboratory. Details of measurements procedure are published elsewhere^38^. EXAFS data was processed using ATHENA and simulated by ARTEMIS. The simulation was performed using quick first shell fit. The water oxidation experiments were performed using an HQ40d portable dissolved oxygen meter connected to an oxygen monitor with a digital readout at 25 °C. Visible spectra were recorded by a mini spectrophotometer (Pooyesh Tadbir Karaneh (Phystec), Iran).

## Synthesis

**1** (Fig. S1, 50 mg) was dissolved in acetonitrile (10 mL). Then, the solution in a crucible was slowly heated for one hour to evaporate the solvent and then it was decomposed at 550 °C for 12 hours in air. The solid was treated by KOH (1.0 M) for 5 hours at room temperature in air.

## Supplementary information

Supplementary Information
